# Chronic Alcohol Ingestion Changes the Landscape of the Alveolar Epithelium

**DOI:** 10.1155/2013/470217

**Published:** 2012-12-30

**Authors:** Charles A. Downs, David Trac, Elizabeth M. Brewer, Lou Ann Brown, My N. Helms

**Affiliations:** ^1^Department of Pediatrics, Emory-Children Center for Developmental Lung Biology, 2015 Uppergate Drive, Suite 316K, Atlanta, GA 30322, USA; ^2^The Nell Hodgson Woodruff School of Nursing, Atlanta, GA 30322, USA; ^3^Emory Alcohol and Lung Biology Center, Atlanta, GA 30322, USA

## Abstract

Similar to effects of alcohol on the heart, liver, and brain, the effects of ethanol (EtOH) on lung injury are preventable. Unlike other vital organ systems, however, the lethal effects of alcohol on the lung are underappreciated, perhaps because there are no signs of overt pulmonary disorder until a secondary insult, such as a bacterial infection or injury, occurs in the lung. This paper provides overview of the complex changes in the alveolar environment known to occur following both chronic and acute alcohol exposures. Contemporary animal and cell culture models for alcohol-induced lung dysfunction are discussed, with emphasis on the effect of alcohol on transepithelial transport processes, namely, epithelial sodium channel activity (ENaC). The cascading effect of tissue and phagocytic Nadph oxidase (Nox) may be triggered by ethanol exposure, and as such, alcohol ingestion and exposure lead to a prooxidative environment; thus impacting alveolar macrophage (AM) function and oxidative stress. A better understanding of how alcohol changes the landscape of the alveolar epithelium can lead to improvements in treating acute respiratory distress syndrome (ARDS) for which hospitalized alcoholics are at an increased risk.

## 1. Alcohol Abuse and Lung Injury

### 1.1. Acute Respiratory Distress Syndrome

It is a serious condition that occurs in critically ill patients alongside infection, injury, and fluid accumulation in the lung that hinders effective oxygen exchange. The role of alcohol abuse in the development of ARDS was established in a clinical study involving 351 medical and intensive care unit patients at university-affiliated city hospitals [1–3]. Based on medical records, the incidence of ARDS was significantly higher in patients reporting a prior history of alcohol abuse (52%) versus patients without a history of alcohol abuse (20%); *P* < 0.001. Moreover, of all patients who developed ARDS, the inhospital mortality rate was 65% in patients with a history of alcohol abuse versus only 36% in patients without a history of alcohol abuse (*P* = 0.003). Several other research groups have likewise reported alcohol-specific lung disease in humans and in animal models for alcohol abuse [4–8].

### 1.2. Alcohol and the Distal Lung

The respiratory zone, where gas exchange occurs, begins when terminal bronchioles lead into respiratory bronchioles and are lined with alveolar sacs. Structurally speaking, the alveolar sacs are made up of alveolar type 1 and type 2 cells covered with interlinking capillaries. Type 1 cells make up greater than 95% of the surface area of the lung, whereas type 2 cells are small and cuboidal; both cell types express functional Na^+^ channels [9, 10]. Because of the rich blood supply to the alveoli, and because alcohol is absorbed and distributed in an unaltered state (unbound to proteins or complexed with other transport systems) it could be argued that the lung is the most vulnerable organ following immediate alcohol absorption. Functionally speaking, alveolar macrophages (AMs) may be considered the third cell type comprising the alveoli (alongside type 1 and type 2 cells). Located near the pneumocytes, albeit separate from the alveolar wall, AMs play a key role in homeostasis, host defense, and tissue remodeling of the lung. Previous studies have shown that chronic ethanol ingestion results in impaired alveolar macrophage function (i.e., decreased phagocytosis and increased reactive oxygen species production) via altered Nadph oxidase activity [11, 12]. Moreover, chronic alcohol abuse also compromises the integrity of alveolar barrier properties [13, 14]; however, there is no overt injury until a secondary injury occurs, such as with sepsis [1]. Because chronic ethanol ingestion impacts all aspects of the alveolar epithelium, it is important to continue to study the consequence of alcohol abuse on all the cells that make up the alveolar airspace. 

## 2. Animal and Cell Culture Models Used to Investigate the Development of Alcohol-Induced Lung Disease

Unlike the adverse effects of alcoholic psychosis, gastritis, and cardiomyopathies, the effects of alcohol abuse on lung injury are not overtly apparent until a secondary injury occurs. As such, alcohol's effect on lung pathogenesis was not explicitly examined in long-term primate studies involving baboons that consumed alcohol with their diets for up to 4 years. It is interesting to note, however, that 3 of the 12 baboons in the longitudinal alcohol study died following upper respiratory infection [15]. Currently, due to constraints associated with cost and manageability, many researchers typically utilize small rodent animal models on either liquid ethanol diets, injections, or EtOH inhalation exposure. Intragastric and intraperitoneal delivery of alcohol (where alcohol is directly infused via a feeding tube or directly injected) indeed elevates blood alcohol content to 230–370 mg/dL but may not be appropriate for modeling lung injury. Laboratory animals treated in this manner primarily develop fatty liver, localized necrosis, fibrosis, steatosis, and inflammation (reviewed in [16]). Chronic and intermittent exposure to alcohol vapor can be effective in elevating blood alcohol levels in the range of 150–250 mg/dL. Recently, inhalation of alcohol vapor has been utilized to effectively study osteoblast proliferation [17]. Generally speaking, however, this approach may be a more suitable model for acute (binge drinking) studies, or behavioral studies, as opposed to alcohol-induced lung injury, given that heterogeneity of alveolar airspaces could limit the reliability of vaporized alcohol delivery in lung studies. Because of these limitations, chronic alcohol abuse and lung injury are usually modeled using liquid diets which allow researchers to control the percentage of alcohol consumption.

### 2.1. Lieber-DeCarli Liquid Ethanol Diet for Rats

 About 30 years ago, Lieber and DeCarli developed nutritionally adequate diets containing ethanol [18]. Today, this diet is still widely used and is available commercially in dry powder from TestDiet, Richmond, IN, USA. When made up, the control diet consists of 19% fat, 15% protein, and 66% carbohydrate. For alcohol feeding, 36% of the total calories as glucose were replaced with an isocaloric amount of ethanol (5 g/100mL). Despite reports of decreased growth rates of rats fed the Lieber DeCarli liquid diets (due to a reduction in the amount of liquid diet consumed), pair wise feeding of experimental groups (where isovolumetric amounts of the same diet with glucose replacing the caloric content of ethanol) can control for weight gain differences arising from aversion to the liquid diets (reviewed in [19]). However, several investigators have been able to successfully model alcohol-induced lung injury using Sprague Dawley rats maintained on the Lieber-DeCarli liquid versus standard chow and water given ad libitum [20–24].

### 2.2. Meadows-Cook Alcohol Mouse Model

 Because the Lieber-DeCarli liquid diet does not completely mimic alcohol and food ingestion patterns, many investigators opt to use what is now termed the Meadows-Cook mouse model [25, 26]. This model initially involves a liquid diet with no ethanol for 3 days, and then the ethanol content is increased by 5% every 3–5 days until the final concentration of 20% ethanol is reached (and is maintained for weeks to months). Maltose-dextrin is typically added to the control diet to account for the calories due to ethanol. This alcohol mouse model is also given standard chow ad libitum. In this way, many investigators have modeled the effects of chronic alcohol ingestion and acute lung injury and obtained blood alcohol levels of 0.08% [12, 27]. Importantly, the Meadow-Cook model opens the possibility of including transgenic mouse animal studies and alcohol exposure. 

**Figure 1:**
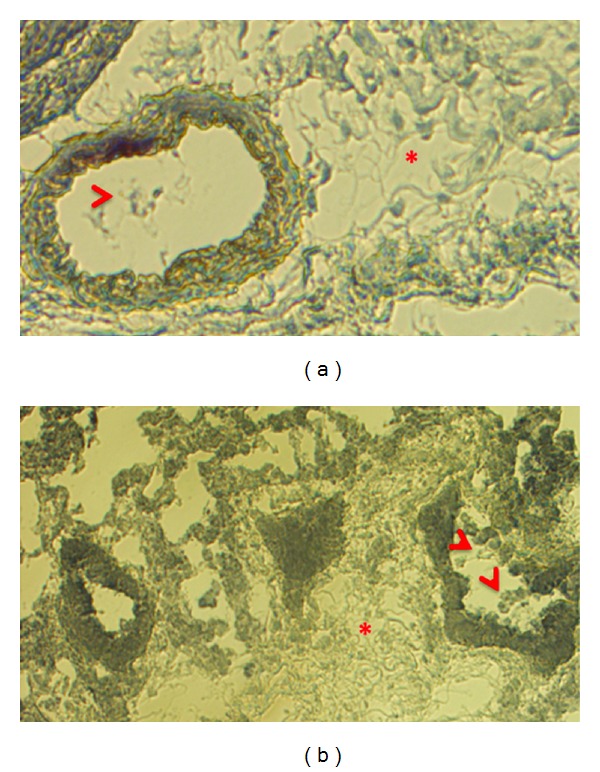
Chronic ethanol ingestion changes the landscape of the alveolar epithelium. 40X original magnification of white light illumination of trichrome-labeled cryoslices obtained from maltoedextrin-fed (control; top) versus chronic EtOH-fed mouse lung (Meadows-Cook model; bottom). (*) indicates inflamed area with exudate. Arrow points to alveolar macrophages inside airway. Note collapsed airway in Meadows-Cook alcohol mouse model versus control-fed animal.

In Figure 1 below, we show cryoslices of lung sample obtained from mice fed a (control) maltodextrin diet and chronic (6 weeks) of 20% w/v Meadows-Cook diet. Tissue samples were subjected to trichrome labeling in order to illustrate changes in the mouse lung following overnight inoculation with 1 mg/mL LPS (in order to model sepsis and ARDS) and chronic alcohol ingestion. The (^∗′^ *s*) indicate exudate and degenerative fiber stains lighter in the EtOH lung versus caloric control in the top and bottom panels. Additionally, the airways are collapsed in EtOH- and LPS-stressed lung, and arrows point to alveolar macrophages with documented dysfunction [11]. 

### 2.3. Tissue Culture Model of the Alcohol Epithelium 

In order to study the effects of alcohol on pneumocytes at the cellular and molecular levels, primary alveolar cells may be isolated (as described in [10, 28]), or alternatively, live lung tissue slices may be obtained from chronic ethanol-fed animals (as described in [29, 30]). Acute effects of ethanol, may be studied via direct application of alcohol to lung epithelial culture medium (maintained in humidified incubators at 37°C and 5% CO_2_) saturated with specified concentrations of ethanol. The fact that alcohol is immediately absorbed following ingestion (unbound to proteins or complexed with other transport systems), and because of the unique network of capillary blood flow into the lungs, direct application of ethanol to the culture medium may well model acute effects of ethanol without concern for alcohol's metabolites, such as acetaldehyde and acetate. 

## 3. Alcohol and Alveolar Epithelial Channel Regulation 

### 3.1. ENaC

 It is a multimeric protein composed of *α*, *β*, and *γ* subunits in a fixed stoichiometry [31, 32]. ENaC activity is the rate limiting step in the resolution of lung edema. The role of ENaC in maintaining a healthy airway epithelium is highlighted by several seminal observations. First, mice lacking the *α*-ENaC subunit die within 40 hours of birth due to an inability to clear lung fluid [33]. Second, electrophysiology studies show that the *α*-ENaC subunit is vital for sodium reabsorption [34]. ENaC can be further classified into highly selective cation (HSC) channels and nonselective cation (NSC) channels according to measurements of unitary conductance and ion selectivity [9]. HSC channels favor Na^+^ reabsorption over K^+^(40 : 1) and have a small conductance (4–6 pS). NSC channels, as the name implies, are less discriminant in Na^+^ over K^+^ selectivity (1.1 : 1) and have a larger unitary conductance (>12 pS) [9]. Newly developed live lung tissue preparations, which enable access to the apical surfaces of both alveolar type 1 and type 2 cells in intact alveolus, have advanced understanding of the biophysical properties of the lung [30, 35]. 

### 3.2. Na,K-ATPase

It is an oligomeric protein composed of *α* and *β* subunits, with the most common combination being *α*1, *α*2, *α*3, or *α*4 with *β*1 (reviewed in [36]). The ATP driven transporter exchanges 3 Na^+^ out of the cell for 2 K^+^ entry into the cell. In this way, the Na,K-ATPase maintains an electrochemical gradient that favors sodium re-entry into cells. In addition to facilitating osmotic movement of ions, recent studies indicate that Na,K-ATPases play additional important roles in maintaining tight junctions, cell polarity, cell movement, and signaling (reviewed in [37]) in epithelia. Homozygous knock-out mice for the three *α* isoforms are embryonic or neonatal lethal; systematic silencing of Na,K-ATPase isoforms caused anxiety-like behavior and compromised locomotor activity, special learning, and memory [38]. 

Because ENaC is located on the apical surface of alveolar type 1 and type 2 cells, it is likely responsible for the rate-limiting step in net salt and water transport in the lung. Vectorial reabsorption of Na^+^ from the alveolar fluid lining generates the osmotic gradient needed to transport water across the epithelium and out of the airspace. We have reported that chronic alcohol ingestion increases ENaC activity in alveolar type 2 cells [20] following 2 and 6 weeks of chronic alcohol consumption. The percent of patches with cation channel activity significantly increased 83% and 78.6%, respectively, in this time frame. However, the precise mechanism of ENaC activation and the impact of ethanol upregulation of channel activity require further investigation. Understanding the signal transduction pathway that alters normal regulation of ENaC in the lung may give rise to novel therapeutic targets for treating chronic obstructive pulmonary disease and addresses why chronic ethanol ingestion predisposes the lung for injury. Because lung fluid volume is central to the pathogenesis of lung injury (reviewed in [39]), we believe that upregulation of sodium reabsorption in the lung initially compounds defects of the alcohol lung. Indeed, airway-targeted overexpression of the *β*-ENaC subunit causes airway surface dehydration, mucus stasis, and inflammation, which favors the growth of bacteria even in germ-free conditions [40–43]. In a related study, albeit distinct study using upper airway Calu-3 cells, Raju and Wang [44] reported that ethanol exposure (25–100 mM) suppresses chloride secretion by CFTR. In terms of vectorial flow of ions, this observation is in line with our observation of net salt and water reabsorption following both chronic ethanol ingestion [20] and acute ethanol application to cells via ENaC stimulation.

Basolaterally, Na,K-ATPases facilitate net solute reabsorption, and there are indeed discrepancies in the literature regarding whether chronic alcohol increases Na,K-ATPase activity [6, 23, 45]. Otis et al. found that alcohol *increased* gene expression of *α*1, *α*2, and *β*1 subunits of Na,K-ATPase and protein expression of the *α*1 subunit [23]. Dada et al., however, found a time- and dose-dependent *decrease* in *α*1 Na,K-ATPase in the alcoholic lung [6]. While these results are directly conflicting, the explanation may lie in how each investigative group modeled chronic alcoholism. Otis et al. placed Sprague Dawley rats on an ethanol diet consisting of 36% of total calories, perfused the lungs after 6 weeks of special diet, and examined protein from whole lung homogenate for *α*1 subunit. Dada et al, on the other hand, treated C57BL/6 mice with 20% v/v daily for 5 days prior to measuring *α*1 Na,K-ATPase in alveolar epithelial cells. Dada et al. found a decrease in Na,K-ATPase using this method and also by treating alveolar epithelial cells with increasing amounts of EtOH (0–100 mM). The discrepancy of findings could be due to the length of alcohol administration. It is possible that the effects of chronic alcoholism on gene expression require a long-treatment window in order to be fully manifested. Despite the differences in reported effect, transport in the lung can be regulated in ways other than channel abundance, and moreover, apical Na^+^ re-entry is believed to be the rate limiting step. 

### 3.3. The Alcohol Lung and Apparent Transport Paradox

 If chronic ethanol consumption primes ENaC activity (with possible facilitation by basolateral Na,K-ATPase transport) and can positively move lung fluid, then why are alcoholics more likely to develop severe ARDS and die? It is not known. However, based on our findings, coupled with published reports, it seems likely that chronic alcohol consumption could lead to dehydration of the epithelial lining fluid—the antioxidant rich fluid which coats the alveolar epithelium. Coupled with secondary injury, such as septic shock, sodium transport, and ENaC expression may be abrogated, and substantial changes in the alcohol-alveolar environment must occur in order to reverse the robust, baseline transport of salt, and water out of the epithelium ([20] and Helms lab unpublished findings) in order for the lungs to flood. Identifying the regulatory agent(s) responsible for reversing the direction of water flow in and out of the lungs is key in effectively treating acute lung injury.

## 4. Alcohol Disrupts Normal Alveolar Barrier Function 

Similar to the plethora of studies reporting intestinal mucosa damage and increased intestinal permeability in humans and animal studies involving chronic ethanol ingestion [46–49], chronic ethanol ingestion also disrupts normal barrier function in the lung. Using radioactively labeled albumin and inulin, Guidot et al. reported that rats maintained on a chronic Lieber-DeCarli liquid alcohol diet had approximately fivefold higher rates of bidirectional protein permeability in the lung (measured in vivo) and about 25% change in basolateral to apical leakage (measured across a monolayer) [20]. In a follow-up study, Fernandez et al. reported that the change in lung permeability may be attributed to a decrease in claudin-1, claudin-7, claudin-18 protein expression, and abnormal accumulation of claudin-5 [14]. Alcohol's effect on claudin expression in the alveolar epithelium is indeed intriguing and differs from the claudin expression profile in Caco-2 cells grown on Martrigel (BD Bioscience) and exposed to ethanol. Elamin et al. reported no change in claudin 2, claudin 4, nor occludin transcript expression levels in ethanol versus control Caco-2 cells [50]. Both groups report ZO-1 mislocalization as probable cause of barrier dysfunction, alongside hyper-*α*-tubulin acetylation, and decreased Nrf2 expression [13, 50]. In two separate studies, the Guidot group successfully restored barrier function in the alcohol lung by repleting antioxidants, such as glutathione in the lung. The beneficial effect of glutathione, foreshadows a proinjury role for ethanol-induced reactive oxygen species in the lung. 

## 5. Alcohol-Induced Oxidative Stress

### 5.1. Alcohol Enhances Nadph Oxidase Generation of Reactive Oxygen Species 

There are 7 members of the Nox family of enzymes that transfer electrons onto oxygen molecules across biological membrane thereby generating reactive oxygen species (ROS) such as O_2_^−^ and H_2_O_2_ (reviewed in [51–55]). The Nox2 isoform is well characterized, is highly expressed in phagocytes, and can be activated upon exposure to microbes, allergens, and inflammatory mediators. Specifically, Nox2 binds to p22phox, which provides a docking site for regulatory subunits (p47phox, p67phox, p40phox, and small G protein Rac1) in a configuration that results in enzyme activation. Generally speaking tissue Nox1-4 isoforms share the same domain structure, and all require p22phox. Nox1-3 are activated by small G protein Rac1 regulatory subunits, whereas Nox4 may be constitutively active or mechanosensitive [56, 57]. Nox5, Duox1, and Duox2 are calcium activated via an EF-hand-calcium-binding domain [53]. The discovery of tissue Nox and Duox isoforms, coupled with their emerging diverse modes of regulation and biological function, has revised oxidative stress and signaling paradigms. Nox-derived reactive species are now widely regarded as signaling molecules; ethanol-mediated activation of Nox enzymes in the lung may significantly alter the landscape of the alveolar epithelium.

### 5.2. The Proinjury Effects of Ethanol Upregulation of Nox Enzymes. 

The role of Nox-derived reactive oxygen species in alcohol lung was described in 2006 by Polikandriotis et al. [24]. Specifically, this group reported increased levels of the Nadph oxidase subunit, gp91phox, using male Sprague Dawley rats maintained on the liquid Lieber-DeCarli diet (containing 36% of calories) for 6 weeks. Since this initial report, we and others have made similar observations in alcohol lung (Helms lab unpublished observations), microglia [58], and in cultured neuronal cells as well as the cerebral cortex of infant mice [59] exposed to alcohol. Interestingly, Yeligar et al. have recently reported upregulation of Nox1, Nox2, and Nox4 expression in alveolar macrophages from alcohol-fed mice and alcoholic patients [12]. 

In lower organisms, cytotoxic bursts of phagocytic ROS may have beneficial effects [51]. In the alcohol lung, however, the consequences of phagocytic recruitment by tissue Nox enzyme may be lethal. First off, ethanol-induced activation of Nox2 turns on ENaC [28]. Hyperactive sodium reabsorption of course dehydrates the luminal surface of the lung which reduces bacterial clearance and increases the resident time of macrophages that are recruited [39]. Because chronic ethanol ingestion increases Nox activity in immune cells, macrophages in the alcohol lung release enormous quantities of reactive oxygen species (and are oxidatively stressed), TGF-*β*1, and IL-13, with significantly decreased phagocytic capacity [11]. This prooxidative environment and mucus stasis (dry lung) occur against a back drop of depleted antioxidant levels of glutathione [20, 21] and zinc [60] in the alcohol lung. Needless to say, the alveolar landscape and immune response in the alcohol lung are severely compromised. 

## 6. Summary 

**Figure 2:**
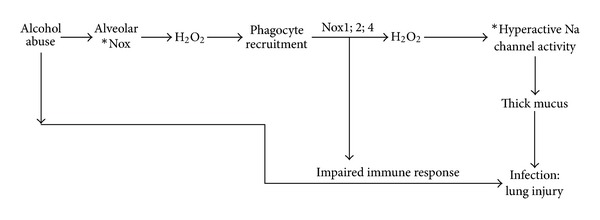
Overview of the complex interplay between cells and proinflammatory mediators which might orchestrate the pathogenesis of acute lung injury. We propose that chronic alcohol abuse disrupts the lung epithelium and activates tissue Nox enzymes, producing low levels of H_2_O_2_, which can diffuse to nearby blood vessels and chemoattract phagocytes. Asterisks indicate causal connection established between alveolar Nox activity and lung ENaC. In the alcohol lung, phagocytes have elevated Nox1, Nox2, and Nox4 activity, which oxidizes the extracellular milieu and alters normal cell function. Because of ongoing drug discovery efforts in developing more sensitive and specific Nox inhibitors, studying the role of Nox enzyme in alcohol-related lung injury is important. Because the alcohol lung is more susceptible to infection and injury, studying the intimate relationship between AM and epithelial transport is important.

Chronic alcohol consumption results in multiple complex changes in the lung. Ultimately, these changes have serious consequences when a second insult, such as pneumonia or sepsis, occurs resulting in an increased incidence of ARDS. Data from our laboratory and others suggest that changes in the alveolar epithelium play a pivotal role in the development of ARDS in alcoholics.  Figure 2 provides an overview of the alveolar landscape and signaling pathways altered by EtOH. Although expected, the precise changes in the alveolar landscape of the alcohol lung postinfection and/or secondary injury remain unclear and are the topic of future investigations.
